# Preliminary experience with a digital robotic exoscope in cranial and spinal surgery: a review of the Synaptive Modus V system

**DOI:** 10.1007/s00701-019-03953-x

**Published:** 2019-05-22

**Authors:** Sajjad Muhammad, Martin Lehecka, Mika Niemelä

**Affiliations:** Department of Neurosurgery, University of Helsinki and Helsinki University Hospital, Helsinki, Finland

**Keywords:** Spinal surgery, Cranial surgery, Digital two-dimensional exoscope, Modus V

## Abstract

**Background:**

The microscope is the standard tool for microneurosurgery worldwide. However, the reduced illumination of the surgical field with the use of a conventional microscope during surgeries of deeply located pathologies remains unaddressed. Accordingly, different exoscope systems have recently been introduced in clinical practice. Here, we report our initial experience with the digital 2-dimensional Synaptive Modus V exoscope system in spinal and cranial surgery.

**Methods:**

During a 2-week period between 27 September to 11 October 2018, we used the Synaptive Modus V exoscope system and performed eight neurosurgical procedures including spine (*n* = 4) and cranial procedures (*n* = 4). Visual quality, depth perception, complications, switching to microscope comfort level of the surgeon, and learning curve during surgery were evaluated.

**Results:**

A total of eight procedures were performed, namely, vestibular schwannoma (*n* = 1), skull base meningioma (*n* = 1), olfactory groove meningioma (*n* = 1), tentorial meningioma (*n* = 1), anterior cervical discectomy (*n* = 1), cervical laminectomy (*n* = 1), and lumbar disc herniation (*n* = 2). The overall results were comparable to the operative microscope with better visual quality and greater comfort for the surgeon.

**Conclusion:**

The Synaptive Modus V system is a safe tool to perform common spinal surgeries and intracranial tumor resection. Image quality is better than a microscope but with slightly less depth perception. Vigorous training in the laboratory may be helpful before clinical use.

**Electronic supplementary material:**

The online version of this article (10.1007/s00701-019-03953-x) contains supplementary material, which is available to authorized users.

## Introduction

The success and safety of a microneurosurgical procedure is heavily dependent on the intraoperative illumination and visualization of pathological tissue and anatomical structures. The introduction of the surgical microscope profoundly impacted the whole field of neurosurgery. The microscope quickly became the core instrument of every neurosurgical operating room. Despite advancements in the functionality of next-generation microscopes, the operating microscope still has some limitations. First, the binocular lenses are attached to the operating microscope and requires the surgeon’s upper body to move along or bend while working at different angles, thus influencing the comfort of the surgeon. Furthermore, illumination during operating in-depth has some limitations that can influence surgical outcome. Tools to improve patient safety, quality of surgical outcome, and surgeon’s comfort are required. Recently, endoscopy was introduced to neurosurgery and quickly broadened its applications. Modern endoscopy has reduced surgical morbidity in certain procedures and improved surgeon comfort. Both 2-dimensional and 3-dimensional exoscopes have recently been introduced in clinical practice in cranial and spinal surgery [1, 3–6]. Most of the current exoscope systems have a strong focus on producing a high-quality 3-dimensional image. However, in addition to a high-quality image, hands-free camera movement is necessary to reduce surgery duration. The Synaptive Modus V system, a 2-dimensional exoscope with a robotic arm and navigated instruments is an excellent approach to simulate hands-free movement of the camera, similar to the mouth switch of a microscope. Here, we report our initial experience with the Synaptive Modus V system in cranial and spinal surgery.

## Methods and materials

We performed a clinical trial of the Synaptive Modus V system in the Department of Neurosurgery, Helsinki University Hospital, during a 2-week period in October 2018. During this time, the Modus V was used in eight neurosurgical procedures (*n* = 4, spine; *n* = 4, cranial). In addition, the system was available for practice on laboratory models outside of working hours. All clinical cases (*n* = 8) were operated on by the second author (ML, senior level neurovascular and skull base surgeon) and assisted by the first author (SM, German board–certified neurosurgeon and Helsinki skull base and vascular clinical fellow). Preoperative testing outside the operating room was performed by ML and SM. Before the clinical trial, ML had a half-day cadaver lab trial with the system about 4 months earlier.

### Exoscope specifications

The Modus V™ is a fully automated, hands-free, robotically controlled digital 2-dimensional exoscope. Tracked surgical instruments provide hands-free robotic movement of the camera and optical focal depth control. The enhanced optics ensure a clear, natural view with × 12.5 optical zoom, < 10 μm resolution, and up to 65 cm working range. There are four enhanced LED light sources surrounding the camera. Combined with a 4K digital medical-grade monitor, Modus V’s cognitive optics™ predict lighting and camera conditions to provide an optimal view of the surgical field.

### Robotically assisted motion

The Modus V has five robotically assisted motions that respond to touch by detecting and performing intended manual adjustments delivering precise visualization. The navigated suction is held in the non-dominant hand and brought to the region of interest. The robotic arm with a camera and light source follows the suction to precisely visualize the point of interest (video 1).

### Surgical overlay

Modus V’s surgical overlay provides real-time, intraoperative feedback on tracking information, optical parameters, and system settings to surgeons while operating.

### Trial setup

During the 2-week trial period, eight procedures were performed (*n* = 4, spine; *n* = 4, cranial). All surgeries were performed by the same team to better evaluate the learning curve and other qualitative parameters. We worked in close collaboration with the manufacturer’s technical assistants who helped in installation of the system, training, and daily setup. With every additional case and the discussions that followed, we streamlined our workflow and devised plans on how to utilize the system even more efficiently. To evaluate clinical use of the Modus V, we quantified visual quality, depth perception, complications, comfort of the surgeon, and surgery duration (Table 1). Utilizing these parameters for a single surgical team, we could evaluate the learning curve of the Modus V in spinal and cranial surgeries.

**Table 1:** Evaluation of the Synaptive Modus V system, a 2D exoscope in clinical practice. The scores for image quality, depth perception, complications, and comfort of surgeon were evaluated as follows: as good as microscope = 0, slightly better than microscope = +, much better than microscope = ++, slightly worse than microscope = −, much worse than microscope = −−, complications: 0 = no complications, + = complication occurred

Case no.	Diagnosis	Procedure	Age (years)	Visual quality	Depth perception	Comfort of surgeon	Complications	Switching to microscope	Operation time (min)
1	Cervical disc herniation C5/6	Anterior cervical discectomy and fusion	53	−	−−	−−	0	No	135
2	Cervical spinal stenosis C5/6	Dorsal laminectomy	68	0	−	−−	+	No	180
3	Vestibular schwannoma	Tumor resection	47	+	−	−	0	No	440
4	Lumbar disc herniation L4/5	Lumbar discectomy	37	0	0	0	0	No	102
5	Anterior skull base meningioma	Tumor resection	49	0	0	0	0	No	100
6	Olfactory groove meningioma	Tumor resection	55	+	0	0	0	No	153
7	Lumbar disc herniation L4/5	Lumbar discectomy	30	0	0	0	0	No	110
8	Tentorial meningioma	Tumor resection	50	++	0	0	0	No	137

## Results

**Fig. 1:**
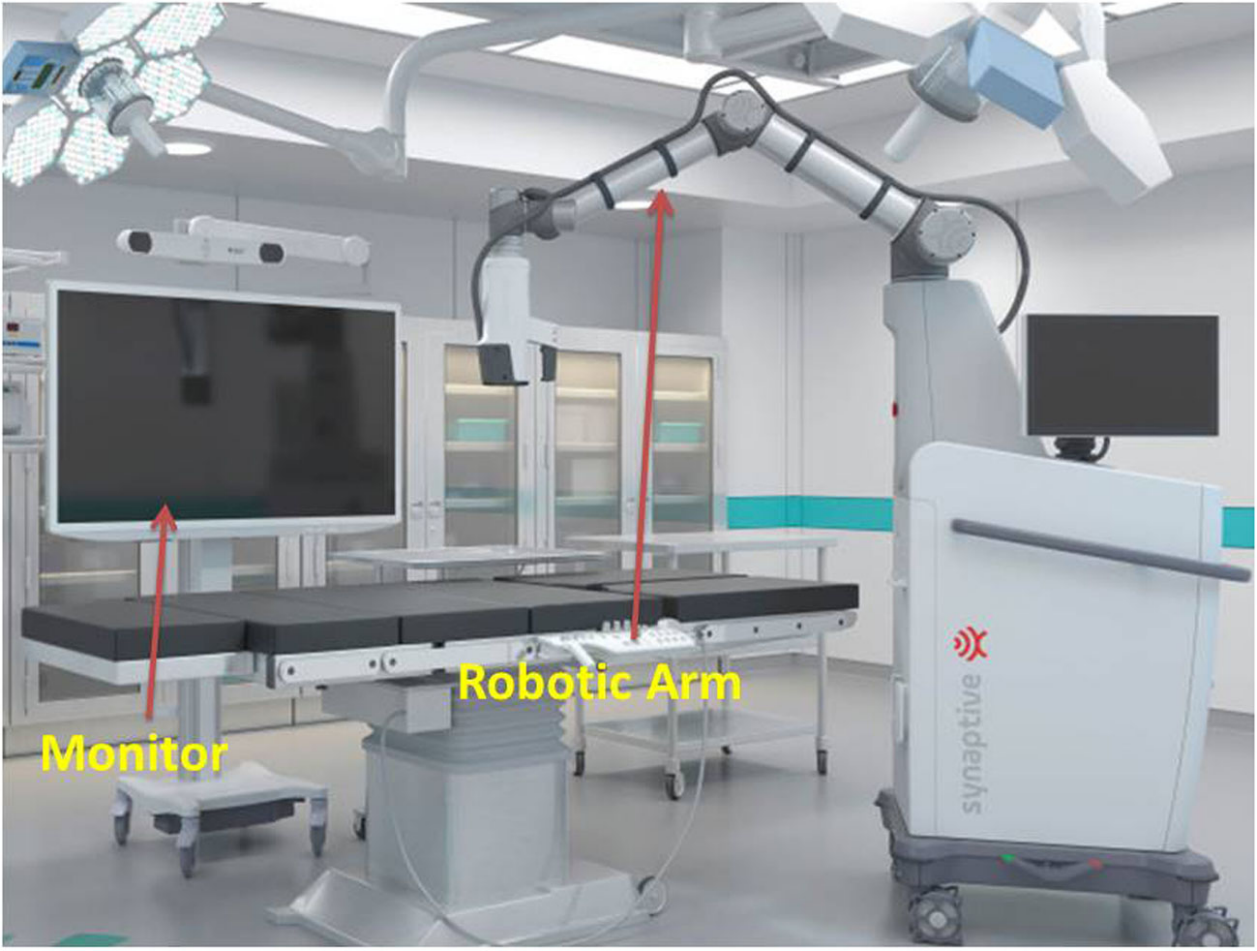
Synaptive Modus V exoscope system

**Fig. 2:**
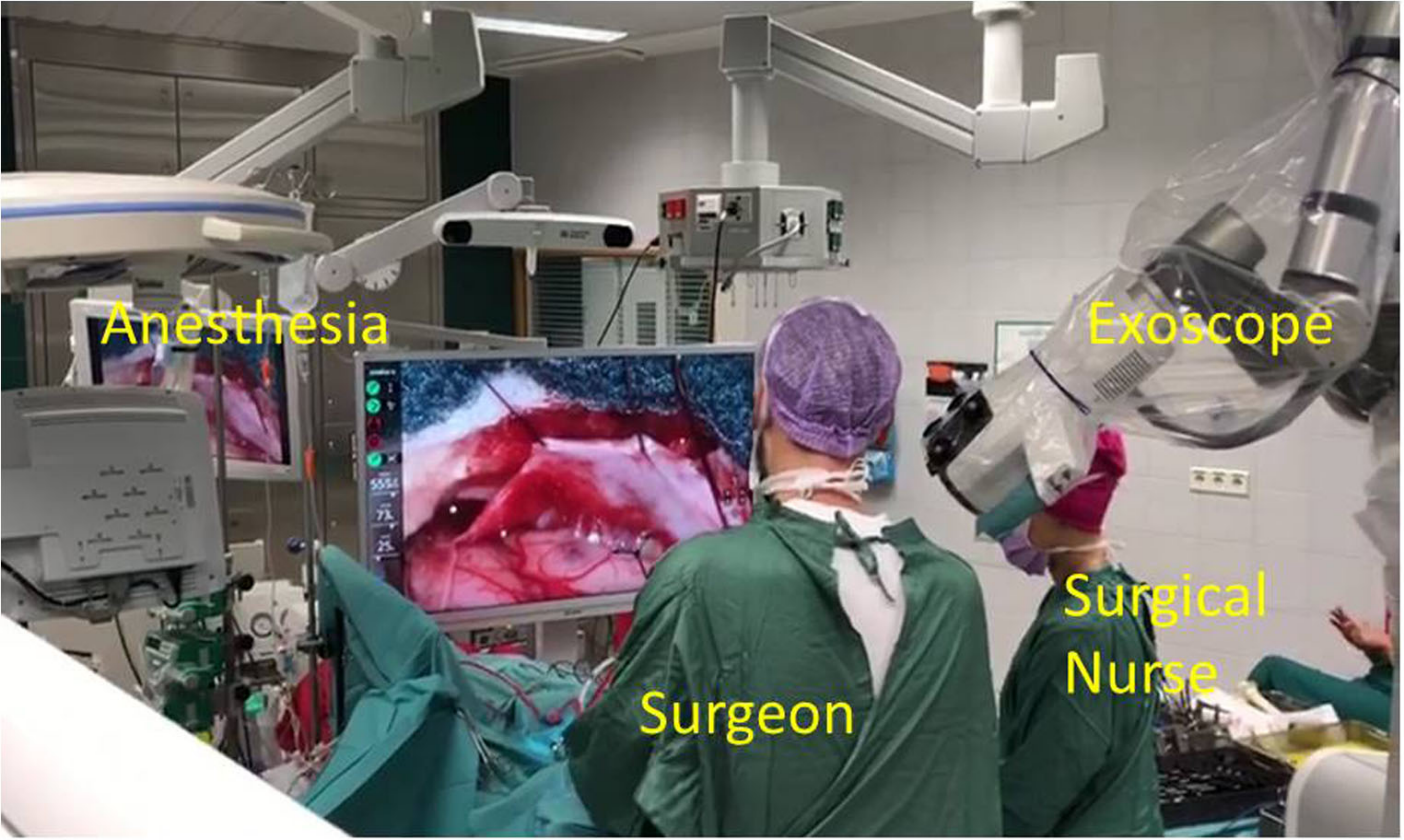
Intraoperative setup for spine surgery

**Fig. 3:**
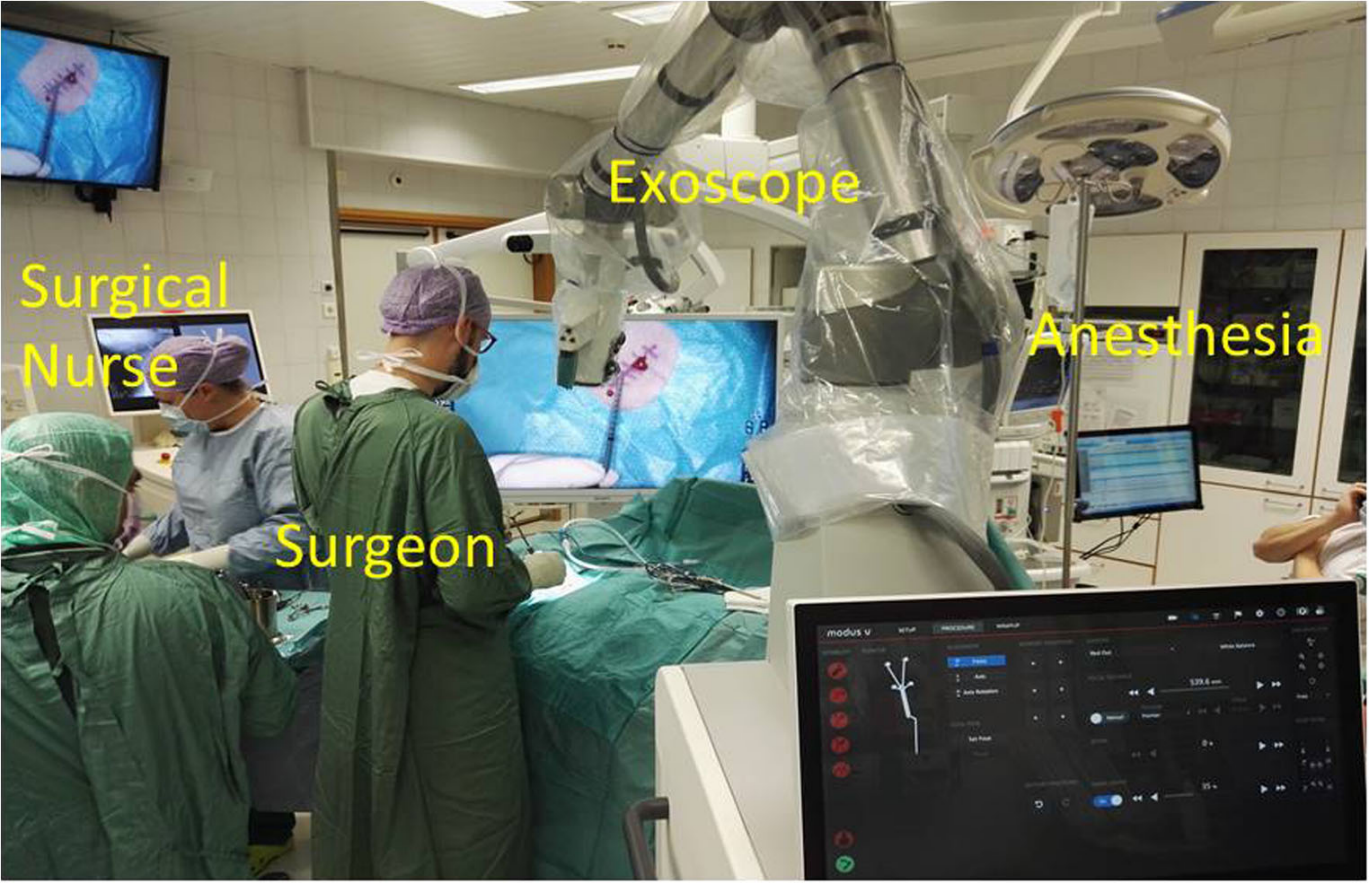
Intraoperative setup for cranial surgery

The overall experience from the clinical trial was positive. The visual quality at depth (image contrast, illumination, and field of view) and surgeon comfort were better than the conventional microscope; the overall depth perception was however poorer (Table 1). The lack of depth perception due to the 2-dimensional camera was the main hindrance throughout the whole trial. This problem was particularly more prominent during the first three surgeries. Over time we adapted to the flat view but this remained the most prominent weakness of the system. Other evaluated parameters were comparable to the conventional microscope (Table 1). The intraoperative setup was maintained in such a manner that we did not experience any major technical problems (Figs. 1, 2, and 3). All patients were operated with the robotic exoscope from beginning to end. We did not have to switch to a conventional microscope at any point. We had a single minor dural tear in the second surgery (cervical laminectomy). There were otherwise no surgical complications. There were challenges but the general performance quality and the results were consistent with what is expected from high-level microsurgery. We operated some easy cases (including cervical and lumbar spine, video 1) but also some complex cases. The difficult surgeries included vestibular schwannoma, pineal region (tentorial) meningioma (video 2), and olfactory groove meningioma. The fact that after only a limited experience with the new system we were able to operate these difficult lesions was very encouraging.

### Operative setup

Several iterations were necessary to identify the settings and workflow that suited us best. Our setup was the following (Figs. 1, 2, and 3 and video 1): (1) screen with a tracking camera directly in front of the surgeon at approximately 2- to 3-m distance, (2) the exoscope mostly on the right side of the surgeon, (3) suction with tracking fiducials in the left hand throughout the whole procedure, (4) camera tracking aligning to the axis of suction, (5) changing suction lengths when needed, (6) autofocus following the tip of the suction, and (7) foot pedal operated by the right foot with buttons (motion activation, zoom in/out, autofocus on/off). We did not use a separate pointer. The suction was used as a pointer throughout the surgery.

## Discussion

Our preliminary experience with the Modus V digital robotic exoscope was encouraging. We performed eight procedures including cranial and spinal procedures; the results were comparable to the operative microscope with better image quality and comfort for the surgeon. The robotic motion of the camera was very precise and allowed seamless working without the need to remove hands from the operative field. This also enabled viewing angles that cannot be achieved with a surgical microscope. The major problem was the 2-dimensional image with lack of depth perception and a significant learning curve in adapting to work style.

### Strengths of the system

The major strength of the Modus V system was the robotic movement of the camera. Even at high magnification (70–100% zoom), the tracking of the suction worked well. Initially, our greatest concern was if we would be able to handle the movement of the camera, particularly in complex skull base tumors. This was not a concern. Rather, the robotic arm provided the advantage of using certain visualization angles that are difficult to achieve with a microscope. Overall, the movement and accuracy of the robotic arm was very good. Tracking of the suction tip for the autofocus purpose worked well. We had the autofocus on for very long periods of time, as it was following the suction tip that was at the point of interest. This technique also helped as a surrogate for depth perception. Work ergonomics were better with the Modus V than with a conventional surgical microscope. The surgeon felt less strain on the neck and shoulder muscles when using the Modus V. This was partially because the surgeon does not need to bite the mouth switch of the microscope, which requires some tension on the jaw and back muscles, and does not need to lean towards the oculars. The draping of the system was easy and the drape stayed securely in place. Image quality and especially contrast and image colors when working at depth were very good. Compared with the direct optical image of a conventional microscope, the image was slightly less sharp at high magnification. However, image contrast and illumination were better at depth. The LED lights, used at a setting of only 5 to 20% and at an offset of 500 to 650 mm, produced much less heat than typical microscope light sources. The color differentiation was good and close to natural.

The intraoperative setup is an important aspect to be discussed. Several iterations were necessary to identify the best possible OR setup (Figs. 1, 2, and 3 and video 1). There is no single standard setup. In our setup, the screen with a tracking camera was directly in front of the surgeon at approximately 2- to 3-m distance, the exoscope mostly on the right side of the surgeon and scrub nurse on the opposite side. The important aspect of the exoscope monitor is that the surgeon, assistant, and scrub nurse all see the same image with the same quality, and the exoscope is not in their way of communication. All scrub nurses who participated in our procedures commented that they felt more involved in the procedure than when using the exoscope, even if they have a similar view from the microscope monitor in front of them.

### Weaknesses of the system

The lack of a 3-dimensional image is the most important weakness of the system. We experienced lack of depth perception not only during the actual surgeries but also during our laboratory sessions. The lack of depth perception made the procedures slower and riskier. The lack of fluorescence modules (such as 5-ALA and ICG) was also a clear disadvantage.

### Recommendations for an effective workflow with the system

The Modus V is not a device that can simply be installed in the operating room and used immediately. The surgeon needs to understand that despite his or her experience, the learning curve is slow. A high level of motivation and a realistic idea about potential difficulties may ease the transition. In our experience, the efficacy in using the Modus V system is related to the time spent using it. With every additional hour and completed case, we became more and more comfortable with it. There are a few key points that may help the surgeon during the use of the Modus V in the operating room. (i) The suction in the non-dominant hand for most of the time is essential for seamless movement of the camera. If one needs to exchange instruments just to initiate movement of the robotic arm, then the biggest strength of the system is lost. (ii) Working under high magnification can achieve optimal visualization of structures. It is necessary to go into relatively high zoom. The image contrast and illumination work best when zoomed in, as reflections from wound borders are limited. (iii) A bloodless operating field is necessary to achieve better contrast between normal and pathological tissue. Taking care of even the smallest bleedings and irrigating the operation field frequently allow for optimal color separation. Especially due to the lack of depth perception, one is completely dependent on textures and colors of structures for recognizing the anatomy.

### Thoughts on clinical trial setup

We made some observations on how to adapt this kind of new technology and how to run a successful trial. First, it is better to have one surgical team perform more procedures than many surgeons performing only a few procedures. It was quite challenging to master the use of the robotic exoscope as the concept of moving and focusing the camera is different from operating a microscope. It took practice and several iterations of settings and working habits before we became comfortable with the system. Second, the trial period should be of sufficient duration to allow adaptation. In our trial, we were much more comfortable with the system during the second week than the first one. Moreover, the 2-week period gave us the opportunity to be more flexible with case planning and to adapt to the new system. Third, before taking the system into the operating room, one should have enough time to work with it on various models. The main surgeon (ML) spent about 10 h of cadaver session practicing with the system before performing the first case. The main surgeon had experience of more than 30 h of working with an exoscope of different manufacturers in a lab setting. Even after this practice, we still felt very uncomfortable during the first surgery. During the first case, we were very close to abandoning the whole trial. Fourth, the initial cases should be carefully selected to be as easy as possible. It is sufficiently challenging to understand how the system works and to adapt to a new concept; any additional stress should thus be limited. Hence, this small number of surgeries may not fully reflect the safety at a larger scale.

### Needs for development

Based on our trial, the most important area for improvement of the Modus V system is a 3-dimensional camera. In addition, florescence modules (5-ALA and ICG) and endoscopic and navigation compatibility will expand the range of procedures. Furthermore, development of tissue recognition features, such as a digital and confocal microscope for pathology, may also be a valuable tool for executing safer surgery. However, at this point, this is much less important than the need for a 3-dimensional view.

### The future of exoscopes in microsurgery

The strengths of exoscopes are the wide field of view and deep focus with good illumination that minimize the need for repositioning and refocusing during the procedure. The published data on the VITOM-3D and ORBEYE exoscope systems report comparable results with the conventional microscope with good image quality and illumination in spine and cranial surgeries [1, 3–5, 7]. Even highly demanding procedures like brain arteriovenous malformation resection and aneurysm clipping have been successfully performed with a 3-dimensional exoscope system [2]. Consistent with the present data, we report similar (and comparable to a microscope) results in our clinical trial with Modus V [1, 3–6].

The current exoscope systems in clinical use are at an initial phase of development but yield results comparable to the modern microscope. The first generation of exoscope systems still has room for improvement. Nevertheless, the novel 3-dimensional high-definition exoscopes with robotic movements and various mechanical designs, based on recent high-tech innovations in digital surgical technology, have set the stage for the next generation in digital image–based neurosurgery.

## Conclusions

The Modus V digital robotic exoscope system is a safe tool to perform common spinal surgeries and intracranial tumor resection. The hands-free movement of the system and image quality was equal to or better than that of a surgical microscope. The main disadvantage of the present system is the 2-dimensional camera with lack of depth perception. Addition of 3-dimensional visualization to the Modus V will be a significant step towards encouraging neurosurgeons to use the Modus V in clinical practice. We recommend training in the laboratory before clinical use.

## Electronic supplementary material

Video 1 Demonstration of robotic arm movements (MP4 95,139 kb)

Video 2 Demonstration video of a spinal and cranial procedure (MP4 69,150 kb)
